# Supplementary material for: A Likelihood Approach for Real-Time Calibration of Stochastic Compartmental Epidemic Models
Source: PLoS Comput Biol. 2017 Jan 17;13(1):e1005257. doi: 10.1371/journal.pcbi.1005257 (PMC5240920; doi:10.1371/journal.pcbi.1005257)
Supplement: S1 File — (TAR.GZ) [file pcbi.1005257.s014.tar.gz › HSPH_Online-SI-Revision/EnKF07/n10-extreme/n10-extreme_EnKF2_table0.pdf]

1	newly 3 weeks spec	724.	98.12	{5., 224.}	87.6492	1.20719	newly 3 weeks cumul	3019.	567.34	{69., 1036.}	82.8917	0.990513	newly 5 weeks spec	306.	38.74	{0., 109.}	88.0915	1.44814	newly 5 weeks cumul	3848.	666.22	{71., 1344.}	84.1357	1.02844
	newly 58 weeks cumul	4437.	753.91	{72., 1696.}	84.2955	1.04286																		
2	newly 3 weeks spec	551.	170.53	{20., 134.}	96.8984	0.964223	newly 3 weeks cumul	1831.	671.05	{153., 514.}	95.9951	0.848922	newly 5 weeks spec	397.	95.23	{3., 98.}	93.3778	1.15247	newly 5 weeks cumul	2702.	890.49	{251., 656.}	94.7561	0.889932
	newly 54 weeks cumul	4210.	1355.36	{273., 1928.}	90.4	0.913544																		
3	newly 3 weeks spec	586.	192.11	{2., 905.}	91.1621	1.35461	newly 3 weeks cumul	2929.	1963.51	{89., 8333.}	104.787	0.855	newly 5 weeks spec	256.	38.82	{0., 84.}	89.3672	1.8695	newly 5 weeks cumul	3549.	2085.49	{94., 8333.}	99.1538	0.890301
	newly 60 weeks cumul	3885.	2144.46	{95., 8333.}	95.6319	0.900796																		
4	newly 3 weeks spec	581.	230.42	{22., 226.}	102.812	1.03512	newly 3 weeks cumul	2166.	815.19	{103., 589.}	100.008	0.961551	newly 5 weeks spec	320.	140.77	{3., 233.}	98.7469	1.09937	newly 5 weeks cumul	2954.	1135.97	{138., 1033.}	99.9665	0.963794
	newly 54 weeks cumul	3942.	1702.81	{207., 3317.}	92.5675	0.921816																		
5	newly 3 weeks spec	669.	66.54	{0., 145.}	94.8221	2.32013	newly 3 weeks cumul	2580.	1523.21	{68., 8048.}	105.217	0.979048	newly 5 weeks spec	386.	43.97	{0., 63.}	97.6295	2.48824	newly 5 weeks cumul	3457.	1619.76	{72., 8048.}	96.6341	1.01975
	newly 57 weeks cumul	4587.	1775.19	{77., 9052.}	91.1988	1.05139																		
6	newly 3 weeks spec	808.	90.66	{4., 207.}	89.5792	1.28952	newly 3 weeks cumul	2954.	533.19	{82., 860.}	83.7539	1.02271	newly 5 weeks spec	362.	36.79	{0., 96.}	90.1243	1.52268	newly 5 weeks cumul	3891.	626.53	{93., 1109.}	85.2231	1.06802
	newly 58 weeks cumul	4944.	711.69	{97., 1390.}	86.478	1.1192																		
7	newly 3 weeks spec	755.	119.39	{0., 183.}	97.4742	1.98534	newly 3 weeks cumul	2728.	1216.48	{49., 3904.}	108.051	1.20297	newly 5 weeks spec	406.	33.01	{0., 27.}	96.6921	2.45763	newly 5 weeks cumul	3682.	1307.08	{50., 3992.}	100.729	1.27285
	newly 59 weeks cumul	4694.	1363.9	{52., 6853.}	96.4499	1.34475																		
8	newly 3 weeks spec	735.	159.76	{18., 171.}	88.2721	1.06448	newly 3 weeks cumul	2632.	1019.83	{248., 1260.}	87.0726	0.744419	newly 5 weeks spec	475.	64.48	{2., 45.}	96.9811	1.616	newly 5 weeks cumul	3719.	1184.86	{263., 1359.}	87.3133	0.837107
	newly 56 weeks cumul	5155.	1284.72	{264., 1384.}	87.4623	0.942971																		
9	newly 3 weeks spec	675.	163.35	{22., 173.}	90.4874	1.03644	newly 3 weeks cumul	2453.	979.4	{224., 1239.}	88.3139	0.749648	newly 5 weeks spec	427.	63.99	{1., 46.}	97.1733	1.56383	newly 5 weeks cumul	3435.	1147.32	{245., 1367.}	89.1994	0.838057
	newly 56 weeks cumul	4640.	1246.93	{254., 1367.}	89.1782	0.932155																		
10	newly 3 weeks spec	697.	170.49	{21., 250.}	88.2023	0.988845	newly 3 weeks cumul	2508.	1044.18	{231., 1422.}	87.3788	0.727083	newly 5 weeks spec	467.	65.22	{2., 77.}	96.1713	1.51123	newly 5 weeks cumul	3582.	1215.27	{256., 1595.}	88.0092	0.81617
	newly 56 weeks cumul	5280.	1320.24	{269., 1629.}	87.8375	0.935421																		
11	newly 3 weeks spec	649.	215.63	{4., 745.}	84.1556	1.20342	newly 3 weeks cumul	2299.	1574.03	{117., 4985.}	85.1192	0.66486	newly 5 weeks spec	447.	63.2	{0., 176.}	90.4072	2.06611	newly 5 weeks cumul	3330.	1756.83	{117., 5000.}	78.4117	0.746086
	newly 51 weeks cumul	4840.	1817.35	{118., 5098.}	73.8936	0.843184																		
12	newly 3 weeks spec	799.	167.1	{26., 223.}	89.8598	1.06698	newly 3 weeks cumul	2755.	801.21	{219., 1113.}	81.9103	0.772983	newly 5 weeks spec	494.	64.27	{1., 86.}	96.2287	1.56746	newly 5 weeks cumul	3861.	966.03	{243., 1257.}	84.4219	0.860914
	newly 56 weeks cumul	5044.	1063.77	{248., 1324.}	85.8888	0.943177																		
13	newly 3 weeks spec	580.	67.38	{0., 76.}	91.4241	1.83926	newly 3 weeks cumul	2137.	825.4	{186., 668.}	91.4712	0.838608	newly 5 weeks spec	319.	7.86	{0., 22.}	97.5361	2.70296	newly 5 weeks cumul	2908.	854.93	{196., 692.}	90.1008	0.942269
	newly 52 weeks cumul	3866.	859.73	{196., 738.}	89.0572	1.04657																		
14	newly 3 weeks spec	614.	65.24	{0., 112.}	94.7915	2.17867	newly 3 weeks cumul	2380.	1644.48	{60., 8752.}	110.272	0.899114	newly 5 weeks spec	361.	44.26	{0., 64.}	97.4183	2.47122	newly 5 weeks cumul	3257.	1741.62	{80., 8752.}	99.0636	0.939367
	newly 57 weeks cumul	4210.	1917.35	{117., 8973.}	92.8886	0.949857																		
15	newly 3 weeks spec	533.	234.27	{20., 193.}	103.139	0.934692	newly 3 weeks cumul	2029.	805.38	{98., 529.}	98.346	0.878763	newly 5 weeks spec	261.	148.25	{6., 226.}	103.398	0.935027	newly 5 weeks cumul	2645.	1141.17	{128., 1019.}	99.5709	0.868797
	newly 54 weeks cumul	3346.	1760.95	{184., 3309.}	94.1097	0.812563																		
16	newly 3 weeks spec	754.	68.64	{0., 110.}	94.8541	2.33001	newly 3 weeks cumul	2774.	1530.33	{61., 7066.}	103.471	0.96959	newly 5 weeks spec	425.	41.23	{0., 52.}	98.1859	2.5637	newly 5 weeks cumul	3788.	1622.12	{71., 7442.}	95.491	1.01929
	newly 57 weeks cumul	4918.	1771.58	{71., 8116.}	90.793	1.04351																		
17	newly 3 weeks spec	715.	130.02	{0., 159.}	99.4853	2.13902	newly 3 weeks cumul	2771.	1110.35	{22., 2548.}	105.494	1.28053	newly 5 weeks spec	457.	39.93	{0., 35.}	97.2363	2.73291	newly 5 weeks cumul	3767.	1218.97	{24., 4293.}	99.9987	1.36247
	newly 59 weeks cumul	4791.	1278.89	{25., 4293.}	96.4377	1.43614																		
18	newly 3 weeks spec	721.	71.4	{0., 134.}	94.8183	2.31076	newly 3 weeks cumul	2736.	1494.46	{85., 7212.}	102.646	0.964037	newly 5 weeks spec	402.	41.9	{0., 58.}	98.1244	2.54099	newly 5 weeks cumul	3695.	1587.59	{87., 7715.}	94.9394	1.00988
	newly 57 weeks cumul	4724.	1738.2	{87., 8247.}	90.5415	1.02728																		
19	newly 3 weeks spec	716.	127.23	{2., 317.}	92.5489	1.54802	newly 3 weeks cumul	3184.	1403.75	{160., 7633.}	94.9777	0.889844	newly 5 weeks spec	379.	30.88	{0., 59.}	96.285	2.34909	newly 5 weeks cumul	4076.	1491.72	{179., 7726.}	90.8008	0.944544
	newly 59 weeks cumul	4764.	1544.02	{188., 7731.}	88.8149	0.986002																		
20	newly 3 weeks spec	765.	166.04	{22., 152.}	92.0261	1.11509	newly 3 weeks cumul	2647.	938.41	{227., 1106.}	87.3453	0.770089	newly 5 weeks spec	468.	57.6	{2., 49.}	97.1368	1.63351	newly 5 weeks cumul	3727.	1090.51	{239., 1176.}	88.4489	0.863097
	newly 56 weeks cumul	5189.	1184.53	{247., 1177.}	88.6959	0.970258																		
21	newly 3 weeks spec	673.	127.53	{0., 239.}	91.5468	2.4661	newly 3 weeks cumul	2256.	1545.36	{534., 3574.}	65.0691	0.384444	newly 5 weeks spec	405.	14.58	{0., 27.}	96.4	2.82516	newly 5 weeks cumul	3166.	1605.85	{540., 3864.}	70.0085	0.498536
	newly 53 weeks cumul	4383.	1612.89	{540., 3898.}	72.7527	0.61045																		
22	newly 3 weeks spec	723.	82.86	{4., 94.}	94.9046	1.615	newly 3 weeks cumul	3471.	738.54	{116., 1166.}	92.2616	1.14846	newly 5 weeks spec	278.	20.77	{0., 18.}	98.1331	2.44196	newly 5 weeks cumul	4204.	798.53	{125., 1536.}	92.5031	1.20991
	newly 60 weeks cumul	4711.	821.94	{132., 1870.}	92.4704	1.25097																		
23	newly 3 weeks spec	587.	121.61	{0., 99.}	103.014	2.30145	newly 3 weeks cumul	1900.	1134.29	{46., 4036.}	101.467	0.9233	newly 5 weeks spec	330.	65.78	{0., 71.}	102.242	2.24782	newly 5 weeks cumul	2657.	1293.73	{54., 4036.}	98.315	0.99054
	newly 55 weeks cumul	3467.	1449.52	{68., 4036.}	94.5798	1.02393																		
24	newly 3 weeks spec	579.	115.85	{0., 321.}	92.3264	2.42376	newly 3 weeks cumul	2054.	1538.54	{431., 2578.}	68.5609	0.394516	newly 5 weeks spec	382.	14.55	{0., 19.}	96.1911	2.8496	newly 5 weeks cumul	2904.	1594.03	{431., 2626.}	72.2655	0.510086
	newly 53 weeks cumul	4101.	1603.33	{431., 2626.}	75.2243	0.632982																		
25	newly 3 weeks spec	727.	178.71	{20., 195.}	91.6575	1.02328	newly 3 weeks cumul	3100.	833.16	{224., 1382.}	81.34	0.8051	newly 5 weeks spec	309.	74.76	{3., 65.}	100.693	1.36705	newly 5 weeks cumul	3847.	1024.56	{242., 1432.}	83.947	0.845335
	newly 56 weeks cumul	4343.	1149.72	{250., 1607.}	85.3332	0.868563																		
26	newly 3 weeks spec	523.	119.35	{0., 89.}	103.776	2.15851	newly 3 weeks cumul	2145.	1051.28	{29., 2322.}	112.453	1.22263	newly 5 weeks spec	188.	36.46	{0., 28.}	103.904	2.34564	newly 5 weeks cumul	2653.	1153.62	{29., 2322.}	108.899	1.26991
	newly 59 weeks cumul	2904.	1201.91	{40., 2322.}	107.597	1.29127																		
27	newly 3 weeks spec	757.	69.3	{0., 126.}	94.8243	2.35239	newly 3 weeks cumul	2773.	1557.04	{53., 6673.}	102.804	0.96846	newly 5 weeks spec	463.	43.57	{0., 62.}	97.9287	2.62072	newly 5 weeks cumul	3879.	1652.09	{63., 7586.}	93.8657	1.02178
	newly 57 weeks cumul	5146.	1815.48	{63., 8067.}	88.8006	1.05268																		
28	newly 3 weeks spec	747.	178.	{1., 788.}	87.4431	1.43533	newly 3 weeks cumul	3109.	1836.9	{113., 5538.}	99.5278	0.878722	newly 5 weeks spec	347.	38.15	{0., 89.}	91.2075	2.02305	newly 5 weeks cumul	3954.	1952.31	{115., 6360.}	93.9122	0.930055
	newly 60 weeks cumul	4582.	2010.82	{115., 7135.}	90.1951	0.960669																		
29	newly 3 weeks spec	724.	99.52	{0., 63.}	96.5718	2.30529	newly 3 weeks cumul	2790.	868.53	{85., 1560.}	87.7387	1.00839	newly 5 weeks spec	343.	69.05	{0., 43.}	101.869	2.17562	newly 5 weeks cumul	3611.	1022.44	{95., 2548.}	87.6356	1.04595
	newly 55 weeks cumul	4378.	1198.12	{100., 2735.}	88.5889	1.06971																		
30	newly 3 weeks spec	685.	106.06	{0., 76.}	97.1416	2.32767	newly 3 weeks cumul	2400.	1156.36	{86., 3219.}	92.1825	0.915918	newly 5 weeks spec	366.	60.31	{0., 33.}	99.6366	2.28418	newly 5 weeks cumul	3210.	1298.72	{113., 4087.}	90.1196	0.959471
	newly 55 weeks cumul	4078.	1456.12	{129., 5207.}	89.2349	0.994134																		
31	newly 3 weeks spec	731.	82.24	{8., 186.}	90.0109	1.23838	newly 3 weeks cumul	2627.	468.71	{74., 795.}	84.7175	1.01216	newly 5 weeks spec	439.	35.52	{1., 95.}	91.9226	1.57804	newly 5 weeks cumul	3631.	556.2	{94., 1031.}	86.163	1.07195
	newly 58 weeks cumul	4700.	644.14	{94., 1290.}	87.2634	1.12401																		
32	newly 3 weeks spec	583.	285.09	{5., 1123.}	91.1098	1.11099	newly 3 weeks cumul	2074.	2165.57	{209., 6491.}	106.535	0.582966	newly 5 weeks spec	399.	44.07	{0., 95.}	90.4887	2.13784	newly 5 weeks cumul	2998.	2320.05	{212., 6921.}	89.2885	0.64255
	newly 50 weeks cumul	4177.	2356.98	{212., 6931.}	77.5753	0.707289																		
33	newly 3 weeks spec	525.	114.93	{10., 254.}	80.3333	0.902358	newly 3 weeks cumul	2485.	669.87	{155., 1306.}	76.0149	0.724035	newly 5 weeks spec	210.	46.76	{1., 141.}	79.1714	1.06936	newly 5 weeks cumul	3022.	788.4	{168., 1703.}	76.2171	0.741062
	newly 58 weeks cumul	3330.	886.49	{168., 2017.}	75.5901	0.741539																		
34	newly 3 weeks spec	536.	256.07	{0., 1004.}	102.614	1.40627	newly 3 weeks cumul	2500.	2001.02	{170., 9612.}	109.566	0.753738	newly 5 weeks spec	238.	72.36	{0., 294.}	91.0924	1.75349	newly 5 weeks cumul	3124.	2211.63	{182., 10157.}	104.048	0.791003
	newly 60 weeks cumul	3558.	2309.72	{187., 10157.}	99.7499	0.810503																		
35	newly 3 weeks spec	625.	96.34	{0., 54.}	99.7216	2.32802	newly 3 weeks cumul	2116.	966.22	{68., 2344.}	95.3516	0.989162	newly 5 weeks spec	382.	60.39	{0., 42.}	100.615	2.31324	newly 5 weeks cumul	2967.	1105.59	{80., 2344.}	93.7664	1.05381
	newly 55 weeks cumul	4257.	1254.84	{89., 2344.}	90.9044	1.1273																		
36	newly 3 weeks spec	695.	109.14	{0., 223.}	90.846	2.50997	newly 3 weeks cumul	2481.	1502.01	{433., 2965.}	65.9609	0.432197	newly 5 weeks spec	436.	10.79	{0., 30.}	97.5252	2.87335	newly 5 weeks cumul	3487.	1549.19	{445., 3177.}	69.9389	0.544324
	newly 53 weeks cumul	5127.	1553.59	{445., 3230.}	75.08	0.687298																		
37	newly 3 weeks spec	692.	135.54	{16., 135.}	91.4191	1.08182	newly 3 weeks cumul	2700.	894.57	{192., 1243.}	87.2026	0.804141	newly 5 weeks spec	388.	41.23	{1., 45.}	95.7191	1.58603	newly 5 weeks cumul	3670.	1006.85	{195., 1302.}	88.1768	0.880261
	newly 56 weeks cumul	4686.	1064.72	{221., 1311.}	88.5907	0.954167																		
38	newly 3 weeks spec	526.	97.03	{0., 126.}	98.9715	1.84498	newly 3 weeks cumul	2700.	1122.38	{87., 1156.}	102.253	1.01718	newly 5 weeks spec	147.	26.65	{0., 35.}	101.952	2.22202	newly 5 weeks cumul	3166.	1197.45	{95., 1226.}	100.121	1.05171
	newly 59 weeks cumul	3369.	1229.23	{97., 1232.}	99.3218	1.06518																		
39	newly 3 weeks spec	662.	103.77	{0., 62.}	101.337	2.38012	newly 3 weeks cumul	2825.	595.34	{64., 466.}	95.2156	1.24103	newly 5 weeks spec	284.	33.82	{0., 23.}	99.9085	2.4425	newly 5 weeks cumul	3546.	690.04	{68., 475.}	95.7665	1.30418
	newly 59 weeks cumul	4001.	734.21	{68., 485.}	95.8338	1.34119																		
40	newly 3 weeks spec	671.	190.18	{21., 295.}	87.7586	0.981992	newly 3 weeks cumul	2384.	1115.08	{215., 2040.}	86.1854	0.704433	newly 5 weeks spec	421.	79.06	{1., 59.}	98.4893	1.54427	newly 5 weeks cumul	3361.	1315.03	{257., 2173.}	87.5802	0.792934
	newly 56 weeks cumul	4464.	1432.34	{261., 2184.}	87.7809	0.877777																		
41	newly 3 weeks spec	690.	76.51	{0., 51.}	96.5522	2.41026	newly 3 weeks cumul	2492.	995.68	{47., 3120.}	89.6966	1.03501	newly 5 weeks spec	409.	50.91	{0., 26.}	99.9878	2.40481	newly 5 weeks cumul	3447.	1110.99	{55., 3136.}	87.6678	1.095
	newly 55 weeks cumul	4485.	1246.69	{57., 3136.}	88.1211	1.15365																		
42	newly 3 weeks spec	566.	117.16	{0., 389.}	86.8021	2.23662	newly 3 weeks cumul	1986.	3039.9	{152., 10965.}	135.202	0.547822	newly 5 weeks spec	394.	19.09	{0., 19.}	99.3173	2.70845	newly 5 weeks cumul	2865.	3099.78	{160., 11121.}	109.666	0.615506
	newly 50 weeks cumul	4248.	3157.98	{160., 11129.}	93.0075	0.706386																		
43	newly 3 weeks spec	779.	63.	{5., 85.}	94.6316	1.6653	newly 3 weeks cumul	3579.	572.23	{73., 692.}	92.0685	1.23553	newly 5 weeks spec	296.	16.35	{0., 14.}	98.5642	2.52378	newly 5 weeks cumul	4369.	620.41	{74., 696.}	92.6173	1.29882
	newly 60 weeks cumul	4830.	640.3	{74., 696.}	92.8646	1.33483																		
44	newly 3 weeks spec	659.	300.64	{20., 944.}	95.736	1.02077	newly 3 weeks cumul	2196.	1478.21	{251., 5303.}	94.3766	0.68538	newly 5 weeks spec	414.	77.91	{1., 154.}	92.7415	1.54597	newly 5 weeks cumul	3184.	1713.28	{258., 6807.}	89.5678	0.767883
	newly 51 weeks cumul	4755.	1804.76	{259., 6812.}	82.1493	0.872038																		
45	newly 3 weeks spec	602.	87.03	{0., 65.}	97.0814	2.309	newly 3 weeks cumul	2275.	992.7	{50., 1676.}	93.2897	0.985834	newly 5 weeks spec	418.	58.96	{0., 35.}	98.3589	2.33838	newly 5 weeks cumul	3189.	1124.62	{69., 3114.}	90.9169	1.04965
	newly 55 weeks cumul	4496.	1289.99	{76., 4933.}	89.1457	1.11999																		
46	newly 3 weeks spec	631.	120.35	{0., 225.}	94.6957	2.5686	newly 3 weeks cumul	2297.	1233.09	{295., 2077.}	69.5921	0.52301	newly 5 weeks spec	464.	12.83	{0., 30.}	97.2349	3.01638	newly 5 weeks cumul	3301.	1288.26	{308., 2108.}	74.5368	0.652127
	newly 53 weeks cumul	4939.	1294.64	{308., 2118.}	78.0814	0.800598																		
47	newly 3 weeks spec	796.	126.86	{0., 105.}	98.2789	2.37044	newly 3 weeks cumul	2859.	1141.33	{81., 3699.}	89.3952	0.975065	newly 5 weeks spec	468.	70.53	{0., 58.}	98.062	2.30792	newly 5 weeks cumul	3902.	1308.84	{99., 3699.}	88.6074	1.02627
	newly 55 weeks cumul	5202.	1464.99	{123., 3799.}	87.1567	1.06967																		
48	newly 3 weeks spec	746.	96.23	{7., 200.}	88.2158	1.185	newly 3 weeks cumul	2942.	565.93	{95., 888.}	82.7107	0.970775	newly 5 weeks spec	435.	39.46	{1., 101.}	90.9287	1.51831	newly 5 weeks cumul	3949.	665.78	{99., 1170.}	84.4852	1.02528
	newly 58 weeks cumul	4973.	754.34	{99., 1398.}	85.7531	1.07271																		
49	newly 3 weeks spec	753.	125.64	{3., 315.}	92.7463	1.4832	newly 3 weeks cumul	3170.	1409.46	{159., 7863.}	95.583	0.890696	newly 5 weeks spec	359.	31.89	{0., 58.}	96.2033	2.32209	newly 5 weeks cumul	4008.	1497.5	{175., 7925.}	91.5429	0.943531
	newly 59 weeks cumul	4662.	1545.63	{178., 7925.}	89.5051	0.983957																		
50	newly 3 weeks spec	849.	65.1	{0., 107.}	95.5548	2.34794	newly 3 weeks cumul	3183.	1476.38	{64., 5411.}	97.3409	1.03851	newly 5 weeks spec	465.	41.33	{0., 58.}	97.9591	2.60596	newly 5 weeks cumul	4282.	1568.03	{83., 6499.}	91.6214	1.08277
	newly 57 weeks cumul	5322.	1730.05	{83., 7934.}	88.5624	1.08548																		
